# Low resolution remote sensing object detection with fine grained enhancement and swin transformer

**DOI:** 10.1038/s41598-025-10286-6

**Published:** 2025-07-07

**Authors:** Zhijing Xu, Xin Wang, Kan Huang, Ren Chen

**Affiliations:** 1 College of Information Engineering, Shanghai Maritime University, Shanghai, 201306 China; 2 State Key Laboratory of Infrared Physics, Shanghai Institute of Technical Physics, Chinese Academy of Sciences, Shanghai, 200083 China; 3 Joint Lab of Calibration and Metrology Technology of Infrared Remote Sensing, Shanghai Institute of Technical Physics, Shanghai, 200083 China

**Keywords:** Low-resolution remote sensing images, Object detection, Fine-grained information, Swin transformer, Shape-IoU, Computational science, Computer science

## Abstract

Object detection in remote sensing images is a highly complex and challenging task. Remote sensing images typically suffer from issues such as small target sizes and densely distributed targets. Existing object detection algorithms often underperform in such scenarios due to their limited capability in handling fine-grained details and multi-scale objects. To address the persistent challenges in remote sensing image object detection, this study introduces a novel detection framework comprising three key innovations. First, we propose the Fine-grained Enhanced Downsampling Network (FEDNet) as the feature extraction backbone, specifically designed to preserve critical target information during downsampling through enhanced fine-grained feature representation. Second, we develop the Swin Transformer-based Progressive Aggregation Network (STPANet), which integrates Swin Transformer Blocks into the C3CST module to achieve superior multi-scale feature fusion while simultaneously capturing global contextual information and local spatial details. Finally, we incorporate the Shape-IoU loss function to optimize bounding box regression, significantly improving small target detection accuracy while maintaining computational efficiency. Experimental results demonstrate that the proposed method achieves outstanding performance on the DOTA and DIOR datasets, with mean average precision (mAP@50) scores of 69.9% and 85.5%, respectively. These results highlight its superior detection performance under low-resolution conditions.

## Introduction

Remote sensing images play a key role in many fields, such as agricultur^1^ urban traffic^2^ animal protection^3^ and ship detection^4^. Due to the inherent challenges posed by remote sensing images—such as complex backgrounds, significant variations in object size, and dense objects—existing detection algorithms often struggle to identify objects quickly and accurately. As a result, there is a pressing need for the development of more efficient detection algorithms.

In recent years, deep learning has become widely adopted in natural language processing and computer vision. Convolutional neural networks (CNNs), in particular, have achieved significant success in object detection and image classification due to their exceptional feature extraction capabilities. Notable CNN-based object detection algorithms include the YOLO serie^5,6^ SSD^7^ and R-CNN^8^ among others. The development and optimization of these algorithms are also closely linked to the availability of high-quality object detection datasets, such as the MS COCO^9^ DOTA^10^ and VOC^11^ datasets.

Current object detection algorithms in the field of remote sensing imagery still face several significant challenges, which stem from various factors. First, remote sensing images are typically captured by sensors or cameras at high altitudes to capture large-scale natural scenes. Unlike ground-based photographs, airborne images offer broad coverage, but their complex backgrounds make object detection more difficult. Second, because remote sensing images are taken from high altitudes, a single image may contain numerous small, irregularly arranged targets, such as ships or vehicles in harbors, which often lack regular patterns. This necessitates algorithms that are particularly adept at detecting small targets^12^. Moreover, remote sensing images often contain objects, such as bridges and overpasses, that exhibit high similarity^13^ increasing the likelihood of omission and misdetection. These characteristics place significant demands on existing object detection algorithms. While current algorithms achieve high detection accuracy, improvements in precision often result in a substantial increase in the model’s parameter count.

Current object detection systems for remote sensing imagery exhibit four fundamental limitations: (1) severe information loss due to aggressive downsampling, (2) constrained receptive fields in feature fusion stages, (3) inadequate joint modeling of global and local contextual information, and (4) suboptimal performance of existing loss functions for small target localization. To overcome these challenges, we propose a specialized detection architecture with the following key contributions:

- Fine-grained Enhanced Downsampling Network (FEDNet) was constructed as the backbone network for feature extraction, replacing conventional convolutional blocks. This network enhances fine-grained information and incorporates an attention mechanism to mitigate channel information loss caused by SPDConv.
- The STPANet was constructed as the feature fusion network. By utilizing Swin Transformer Blocks to build the CST module, the efficiency of feature fusion was significantly enhanced.
- The Shape-IoU loss function was employed to replace the original CIOU loss function in the detection head, thereby improving the localization accuracy for small targets.

## Related work

### Traditional Object detection in remote sensing images

Before the advent of deep learning, or in its early stages, target detection algorithms relied heavily on manual feature extraction. Due to limitations in early image coding, traditional detection methods required complex feature extraction techniques and various optimization strategies to align with available computational resource constraints. For instance, methods such as Histogram of Oriented Gradients (HOG)^14^ and Scale-Invariant Feature Transform (SIFT)^15^ demanded substantial time and human resources for data processing.

### Object detection based on deep learning method in remote sensing images

Due to the limitations of traditional object detection algorithms and the rise of deep learning, deep learning-based object detection algorithms for remote sensing images have become a major area of research. Unlike traditional methods, these algorithms leverage the powerful feature extraction capabilities of neural networks to improve both the accuracy and speed of object detection in remote sensing images, while also reducing human effort.

Deep learning-based object detection algorithms can be broadly classified into two categories. The first category includes single-stage object detection algorithms, such as the YOLO^5,6^ series and SSD^7^. The SSD framework achieves enhanced detection accuracy through its multi-scale feature map approach, albeit at the cost of reduced processing speed. In contrast, YOLOformulates object detection as a regression problem, prioritizing computational efficiency and real-time performance while exhibiting marginally lower precision metrics. Among the various versions of the YOLO series, YOLOv5^16^ strikes a balance between performance and speed. The second category consists of two-stage object detection algorithms, which rely on candidate regions, such as R-CNN^8^ Faster R-CNN^17^ and SPPNet^18^.

The YOLO object detection model has gained significant attention since its inception. YOLOv2^6^ introduced joint training to accelerate model inference. YOLOv3^19^ leveraged the strengths of ResNet^20^ and proposed Darknet-53. YOLOv4^21^ further optimized the Darknet-53 module by integrating CSP. Additionally, the spatial pyramid pooling (SPP) module was incorporated into the backbone network. YOLOv5^16^ achieves excellent performance in natural image object detection tasks by utilizing CSPDarknet as the backbone for feature extraction, PANet^22^ as the neck layer network structure, and introducing the new SPPF module.

R-CNN^8^ first generates a set of region proposals using selective search, and then AlexNet^23^ is used to extract convolutional features for each proposal. A Support Vector Machine (SVM)^24^ is then applied to these features to classify the target category in each proposal. However, due to its slow processing speed, Fast R-CNN^25^ was developed as an improvement. Fast R-CNN processes the image through the CNN only once to extract the feature map. Faster R-CNN further improves efficiency and accuracy by replacing selective search with a Region Proposal Network (RPN) to generate high-quality proposals.

To address the challenges of remote sensing images with complex backgrounds, large variations in target sizes, and overlapping targets, researchers have proposed numerous innovative techniques. Mashformer^26^ is a hybrid detector that integrates CNN and Transformer models, leveraging their ability to capture multi-scale features, which strengthens its representation of complex backgrounds. Li^27^ was the first to introduce large selective convolutional kernels in remote sensing image object detection, proposing the lightweight LSKNet framework, which dynamically adjusts the spatial sensing field to enhance discriminative ability in complex backgrounds. Gao^28^ designed FSOD4RS to sovle the problem caused by the large variation of the target size. Hong^29^ Improved YOLOv3 for ship detection: optimized anchor boxes via k-means++, Gaussian uncertainty for localization, and four anchors per scale for size/orientation variations. Wan^30^ developed a multi-detector head YOLOv5 that incorporates a hybrid attention mechanism at the Backbone layer. Gong^31^ improved this model by proposing AFADet, an adaptive feature-awareness detector capable of real-time, high-accuracy detection of remotely sensed images.

Xu^32^ introduced HODet, which solves the performance degradation caused by object detection due to HBB by predicting the rotation Angle. Ge^33^ proposed YOLOX, an anchorless method that decouples the detection and classification heads, relying on deformable convolutions to capture target-relevant features and reduce misjudgments. Cheng^34^ developed MFANet, a YOLOX-based multi-feature fusion and attention network that enhances feature extraction for targets of various sizes, improving detection accuracy through reparameterization, multi-branch convolution, and attention mechanisms, as well as loss function optimization. Although these methods eliminate anchor points, they still involve complex post-processing techniques for key point matching. FE-CenterNet^35^ incorporates feature aggregation structure (FAS) and an attention generation structure (AGS) into CenterNet^36^improving the ability to perceive small objects. DRADNet^37^ is a method based on degenerate reconstruction, which introduces a hybrid parallel attention feature module to improve the detection accuracy in the case of low and medium resolution. The improved CycleGAN^38^ model achieved promising results in detecting small targets. He et al. introduced the Adaptive Unsupervised Transformer (AST) for optical remote sensing image detection, which learns multi-scale semantics through mask image modeling during pre-training, releasing valuable supervised signals from a large corpus of unlabeled remote sensing images. Finally, Zhu^39^ proposed TPH-YOLOv5, which replaces the original detector head with the TPH detector head, achieving excellent results in dense object detection scenarios.

While current object detection algorithms demonstrate significant performance improvements, these advances typically come at substantial computational costs: reduced inference speeds and dependence on high-resolution inputs. This presents particular challenges for remote sensing applications, where images often have limited native resolution. Moreover, processing high-resolution aerial imagery imposes prohibitive GPU memory requirements and significantly extends training durations, making such approaches impractical for resource-constrained systems. To address these limitations, our work proposes a resolution-agnostic framework that achieves comparable accuracy to high-resolution models while substantially reducing both memory consumption and training time, enabling effective deployment on low-end hardware.

Dong^65^ proposed a Transformer-based fine-grained object detection method, which significantly improves the accuracy of directional object detection in remote sensing images through a new attention mechanism, multi-scale fusion, and optimized classification loss function. Pu^66^ proposes a novel parameter efficient fine-tuning algorithm named Lora-Det. By introducing LoRA modules into the Transformer backbone network and the detection head, and combining the low-rank approximation method with the hybrid fine-tuning strategy, it achieves close to full fine-tuning performance with a small number of parameter updates. Chen^67^ proposed a remote sensing object detection algorithm called K-CBST YOLO, which significantly improves the detection accuracy of small objects in complex background by combining CBAM and Swin-Transformer to enhance the global semantic understanding of feature maps. Ma^68^ proposed FGIR based on Swin Transformer. The architecture generates an attention map of recursive product by integrating the attention weights of local Windows, and tracks the changes of attention weights to enhance the feature extraction effect. Xu^69^ proposed a Transformer-based UAV target detection model. By designing the foreground-enhanced attention Swin Transformer framework and an improved weighted bidirectional feature pyramid network, the detection accuracy of dense small targets was effectively improved. This study and the above five methods also all use Swin Transformer and use fine-grained enhancement module, but the difference is that this study achieves model performance improvement with smaller Gflops and parameters.

### The attention mechanism

The attention mechanism, inspired by human visual attention, is widely utilized in deep learning to improve the model’s ability to focus on important information within the input data. In the field of natural language processing (NLP)^40^ it primarily establishes shortcuts between the context and input, enabling the model to focus on different parts of the data. Biformer^41^ propose a dynamic sparse attention mechanism, implemented through two-layer routing, for flexible computation allocation and content-aware. Sea-Former^42^ design a generic attention block characterized by the formulation of squeeze Axial and spatial enhancement. Efficient Multi-Scale Attention Module (EMA)^43^ focus on retaining the information on per channel and decreasing the computational overhead. EMA groups the channel dimensions into multiple sub-features and makes the spatial semantic features well-distributed inside each feature group. Agent Attention^44^ balances computational efficiency and representation power by introducing a set of proxy tokens. The proxy token acts as a proxy for the query token, aggregating information from key-value pairs and then broadcasting the information back to the query token, significantly improving the computational efficiency. Histoformer^45^ improves on Transformer^46^ and proposes a histogram self-attention mechanism, which divides spatial features into buckets according to intensity, and applies self-attention between or within buckets to selectively focus on similar degraded pixels within a dynamic range. Differential Attention^47^ filters out common mode noise by adding a Softmax in the attention layer and then subtracting two Softmax, so that attention can focus more on relevant content. CNNs excel at feature extraction in local regions, while Swin Transformer^48^ is effective for capturing global features.

## Method

### Proposed method

**Fig. 1:**
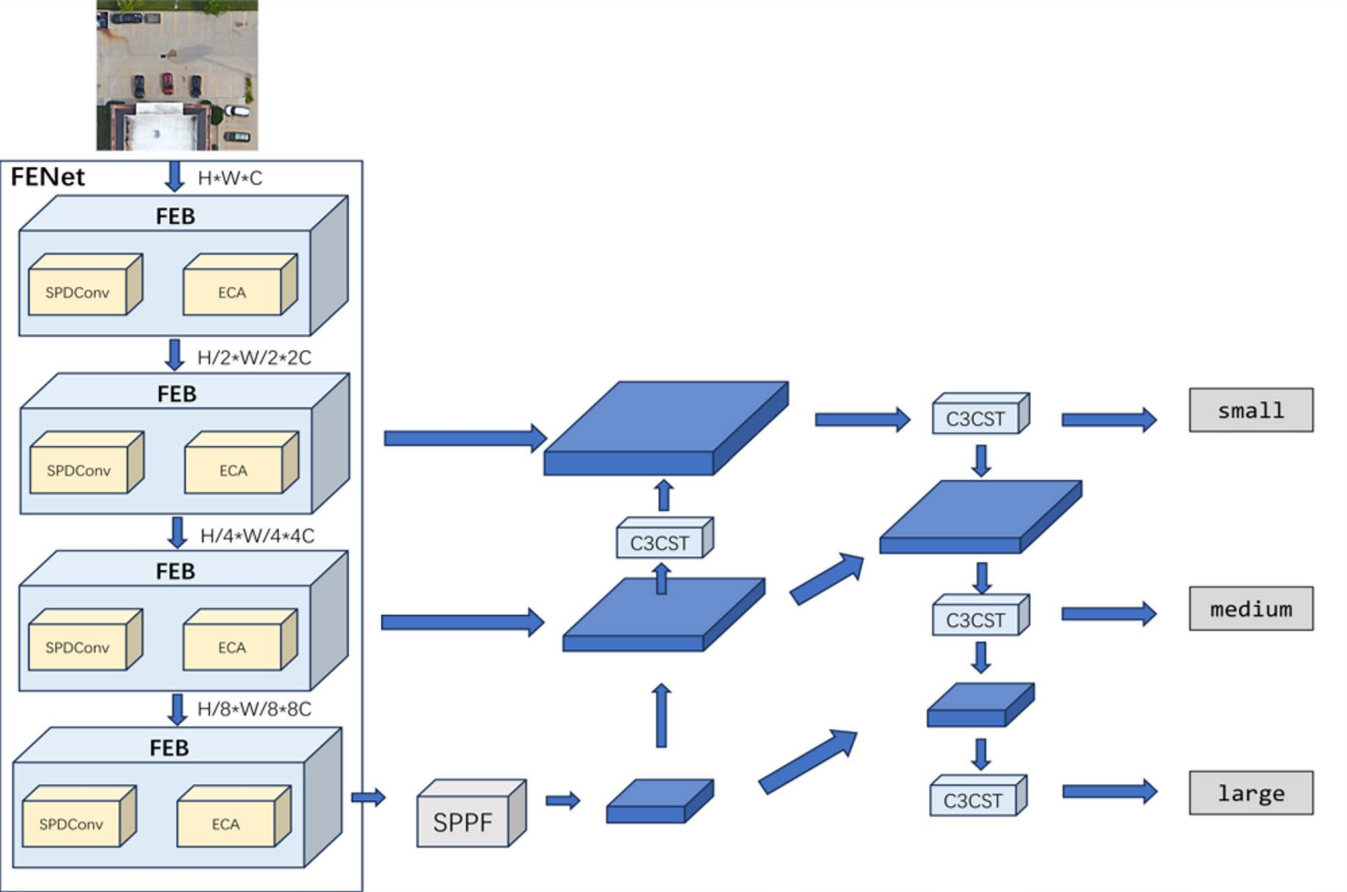
Overall architecture of the proposed remote sensing object detection model.

The proposed object detection framework for high-resolution remote sensing imagery is schematically illustrated in Fig. 1. The model comprises three main components: the Fine-grained Enhanced Downsampling Network (FEDNet), the Swin Transformer-based Progressive Aggregation Network (STPANet), and a detection head that incorporates a customized loss function designed to improve detection accuracy.

First, the preprocessed remote sensing images are input into FEDNet, which consists of four Fine-grained Enhancement Blocks (FEBs). FEDNet enhances fine-grained feature details while concurrently reducing spatial dimensions. The resulting feature maps are then processed by the Spatial Pyramid Pooling Fast (SPPF) module to accelerate processing and maintain effective multi-scale feature fusion.

The feature maps are subsequently processed by an improved Path Aggregation Network (PANet). Initially, the top-down pathway integrates high-level semantic features with low-level spatial features, enriching the representation of target objects. Subsequently, the bottom-up pathway further reinforces low-level localization cues. A sliding window attention mechanism is incorporated to strengthen contextual understanding. This design enhances the model’s ability to extract multi-scale features effectively, thereby improving detection accuracy for targets of varying sizes.

Finally, the detection head receives the feature maps and applies the Shape-IoU loss function, improving detection accuracy by better aligning predicted and ground-truth shapes. This integrated architecture ensures robust and accurate detection across a wide range of object scales and image resolutions.

### Fine-grained enhanced downsampling network (FEDNet)

**Fig. 2:**
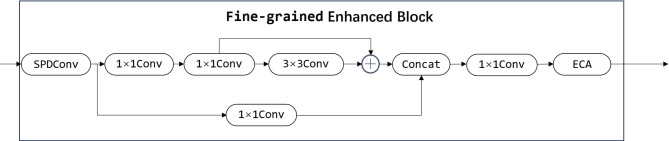
Structure of the Fine-grained Enhancement Block in FEDNet.

The Fine-grained Enhanced Downsampling Network (FEDNet) consists of four concatenated Fine-grained Enhancement Blocks (FEBs), designed to enhance detailed feature representations while simultaneously performing downsampling. The architecture of FEDNet is illustrated in the left panel of Fig. 1. Each of the FEB is composed of SPDConv, Conv, and ECA. Initially, SPDConv processes the input feature map $X \in {{\mathbb{R}}^{H \times W \times C}}$ into $X^{\prime} \in {{\mathbb{R}}^{\frac{H}{2} \times \frac{W}{2} \times 4C}}$. Compared to conventional convolutional blocks, SPDConv is capable of preserving more fine-grained information in the feature maps during downsampling. Subsequently, the feature maps are passed through two parallel branches. The first branch undergoes deep feature extraction via a 1 × 1Conv layer followed by a residual module. The residual module is employed to replace multiple Conv layers, thereby enhancing computational efficiency. The residual module contains a 1 × 1 Conv layer and a 3 × 3 Conv layer. The 1 × 1 convolution is used for channel dimensionality reduction, which helps the 3 × 3 convolution to reduce the operation operation. The 3 × 3 convolution is suitable for extracting local features, and its step size of unity will not change the feature map size. The second branch simply passes through a single 1 × 1Conv layer. Then, the features from the two branches are fused, and then through a 1 × 1 convolutional layer, the channel is compressed to get $X^{\prime\prime} \in {{\mathbb{R}}^{\frac{H}{2} \times \frac{W}{2} \times 2C}}$. The resulting feature map $X^{\prime\prime\prime}=\alpha \times X^{\prime\prime}(\alpha =\sigma (Conv1{D_k}(z)))$ then processed by the Efficient Channel Attention (ECA) module. The ECA module is designed to alleviate channel information loss resulting from aggressive channel compression. The FEB reduces the spatial dimensions of the feature maps while increasing network depth, expanding the receptive field, and enhancing fine-grained feature details. The structure of the FEB is illustrated in Fig. 2. The subsequent section provides a detailed explanation of the components and functions of SPDConv and ECA.

**SPDConv**.

According to previous studies, increasing the depth of a network can lead to the loss of fine-grained details, particularly for small targets. To address this issue, this study employs SPDConv^49^ as a replacement for conventional downsampling convolutional layers. SPDConv enhances the ability to preserve small target information during the downsampling process, thereby improving the performance of the object detection model for remote sensing images.

SPDConv involves two steps. First, assume that the size of any object is ${{\mathbb{R}}^{S \times S \times C}}$. Given that the feature mapping is $Z$, the sub-mapping ${f_{x,y}}$ comprises all constituent elements $Z(i,j)$ i.e., $i+x$ and $i+y$ are divisible by integer proportions.

**Fig. 3:**
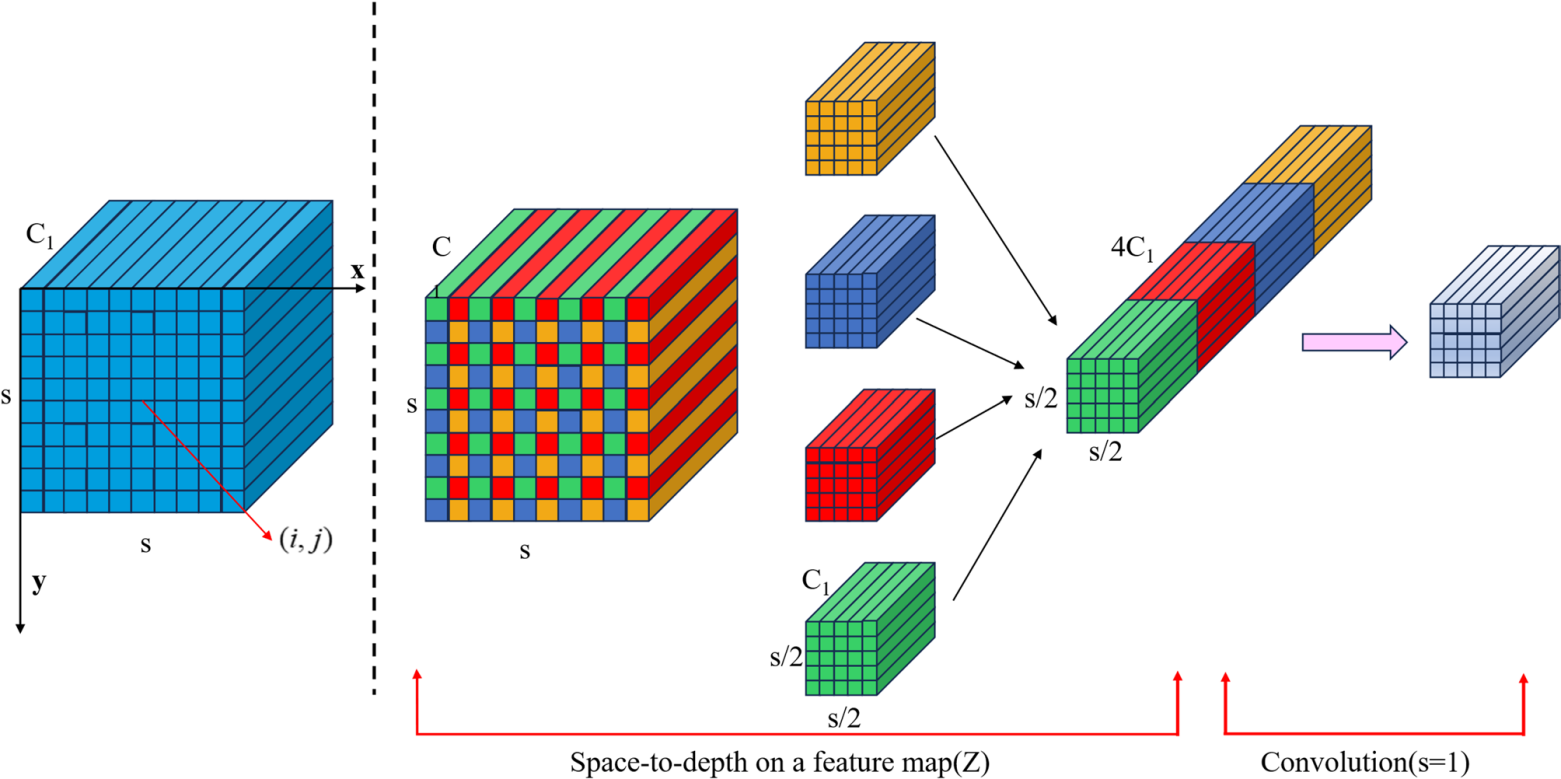
Schematic of SPDConv with a scale factor of 2.

Each word feature reduces the sample $X$ by a scale factor corresponding to its dimension. This process yields four sub-features ${f_{0,0}},{f_{0,1}},{f_{1,0}},{f_{1,1}}$, and each dimension becomes $(\frac{S}{2},\frac{S}{2},{C_1})$. These sub-features are then concatenated along the channel dimension to obtain a feature map $Z^{\prime}$. Its spatial dimension is reduced by the scale factor, while the channel dimension is increased by the square of the scale factor to obtain $Z^{\prime}(\frac{S}{{scale}},\frac{S}{{scale}},scal{e^2}{C_1})$. Figure 3 illustrates the structure of SPD-Conv when the scale factor is set to 2.

### ECA

**Fig. 4:**
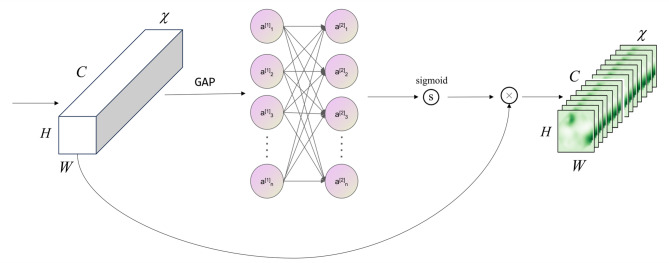
Architecture of the Efficient Channel Attention module.

In CNNs, the attention mechanism has become a key technique for enhancing model performance. Efficient Channel Attention (ECA)^52^ improves the network’s feature representation capabilities due to its efficiency and adaptive properties. ECA primarily functions to strengthen the model’s focus on important features, thereby enhancing its performance across various visual tasks. By incorporating ECA, the model can assign different output weights to each channel, enabling the network to concentrate on features that are most relevant to the task. Figure 4 illustrates the structure of ECA.

ECA captures channel dependencies through one-dimensional convolution. Excessive dimensionality operations can lead to information loss. In contrast to SENet, ECA avoids such dimensionality changes to preserve information while minimizing additional computational overhead. The ECA module first adaptively calculates the kernel size $k$ of the one-dimensional convolution based on the number of channels $C$ of the input feature, with the following formula:

1 $$\left[ {k={{\left| {\frac{{{{\log }_2}(C)}}{\gamma }+\frac{b}{\gamma }} \right|}_{odd}}} \right]$$

where $C$ represents the number of input channels, $\gamma$ and $b$ is the hyperparameter. We take $\gamma =2$ and $b=1$ in the study.

After obtaining $k$, the importance of each channel relative to the others is learned, which can be expressed as:

2 $$\left[ {out=\sigma (Conv1{D_k}(\delta (L))} \right]$$

$\sigma$ is the Sigmoid activation function, $Conv1D$ is a one-dimensional convolution operation with kernel size to capture inter-channel dependencies. $\delta (L)$ represents the result after global average pooling, compressing the feature map $L$ to the size of${{\mathbb{R}}^{1 \times 1 \times C}}$.

Finally, the calculated attention weight $out$ is multiplied element-by-element back to the original feature map $L$ to complete the adjustment of the importance of different channels:

3 $$Output=out \odot L$$

$\odot$ means element-wise multiplication.

SPDConv expands the channel dimension by a factor of four. To mitigate potential information redundancy, a 1 × 1 convolutional layer is employed for channel dimensionality reduction. However, this operation inevitably leads to the loss of channel information. To mitigate this issue, we incorporate the ECA module to reduce channel information loss. Subsequent experiments have demonstrated the effectiveness of the ECA module in preserving critical channel information.

### Swin Transformer-based progressive aggregation Network(STPANet)

**Fig. 5:**
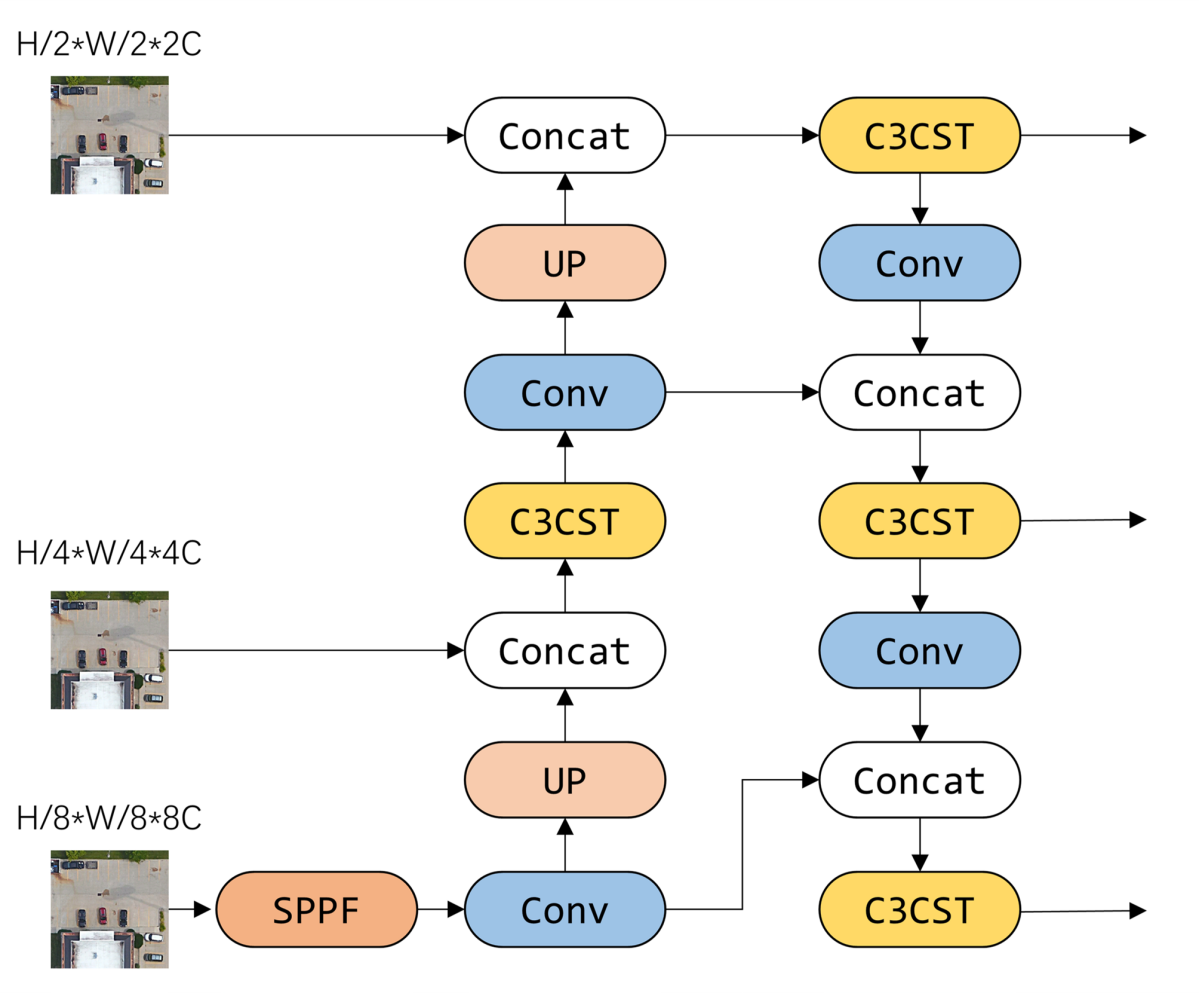
Network architecture of STPANet.

Shallow networks are well-suited for capturing the positional information of small targets, whereas deeper networks excel at extracting semantic information from medium and large targets. Motivated by this observation, the Swin Transformer-based Progressive Aggregation Network (STPANet) was designed. The structure of STPANet is illustrated in Fig. 5. STPANet enhances feature fusion within PANet by integrating Swin Transformer Blocks, thereby improving global-local context modeling for multi-scale object detection.

The original PANet first utilizes a top-down pathway, where feature maps are upsampled by a factor of two and fused with same-resolution features from FEDNet, thereby combining high-level semantic information with low-level spatial features. A subsequent bottom-up fusion stage reinforces low-level localization cues. To build upon PANet, the proposed STPANet integrates CST module after the feature fusion process. The Swin Transformer Block within the CST module enhances contextual representation and provides effective multi-scale feature extraction, improving detection accuracy across objects of various sizes. Additionally, the CST module mitigates the vanishing gradient problem and improves training efficiency and stability.

STPANet primarily employs the CST module to incorporate the capabilities of the Swin Transformer and replaces the original C3 module with a modified version, C3CST, which integrates the CST module. This design enables STPANet to incorporate both window-based and sliding window attention mechanisms for enhanced feature extraction. The structures of CST and C3CST are described in detail in the following section.

From the flow of Fig. 5, we can know that firstly, feature map $Y \in {{\mathbb{R}}^{\frac{W}{8} \times \frac{H}{8} \times 8C}}$ is extracted by a 1 × 1 channel convolution and channel compression to obtain $Y^{\prime} \in {{\mathbb{R}}^{\frac{W}{8} \times \frac{H}{8} \times 4C}}$. Then$Y^{\prime} \in {{\mathbb{R}}^{\frac{W}{8} \times \frac{H}{8} \times 4C}}$ is upsampled and concatenated with $F \in {{\mathbb{R}}^{\frac{W}{4} \times \frac{H}{4} \times 4C}}$ to get $Y^{\prime\prime} \in {{\mathbb{R}}^{\frac{W}{4} \times \frac{H}{4} \times 8C}}$. Finally, $Y^{\prime\prime\prime} \in {{\mathbb{R}}^{\frac{W}{4} \times \frac{H}{4} \times 4C}}$ is obtained by feature extraction through C3CST. After repeating the above operations, the corresponding feature maps of three sizes are obtained respectively, and these three size maps are sent to the detection head.

### C3CST

Swin Transformer is a variant of Vision Transformer, offering several advantages, such as lower computational complexity, increased efficiency in processing high-resolution images, and the ability to combine local and global feature extraction. Therefore, we utilize its window attention and sliding window attention mechanisms to create the new C3CST module.

The most important module in the Swin Transformer Block is Window Multi-Head Self-Attention (W-MSA), which primarily serves to reduce computational complexity. In contrast to the Multi-Head Self-Attention (MSA) in Vision Transformer, which calculates self-attention for each pixel in the feature map, W-MSA only computes self-attention for each window after partitioning the feature map into chunks. The following equation illustrates the difference in computational complexity between the two modules.

4 $$\Omega \left( {MSA} \right)=4hw{C^2}+2{\left( {hw} \right)^2}C$$

5 $$\Omega \left( {W - MSA} \right)=4hw{C^2}+2{M^2}hwC$$

$h$, $w$ and $C$ represent the height, width and depth of the image, respectively. $M$ denotes the window size.

The formula expression for self-attention is shown below:

6 $$Attention\left( {Q,K,V} \right)=SoftMax\left( {\frac{{Q{K^T}}}{{\sqrt d }}} \right)V$$

Where $Q$ stands for Query,$K$ for Key, $V$ for Value, $Q,K,V \in {{\mathbb{R}}^{{M^2} \times d}}$, $d$ is querykey, and ${M^2}$ is the number of patches, usually 9. $SoftMax\left( {\frac{{Q{K^T}}}{{\sqrt d }}} \right)$ is an attention feature matrix that represents the relationship between all elements and other elements in the map. $Attention\left( {Q,K,V} \right)$ aggregates global information.

Since Window Multi-Head Self-Attention (W-MSA) is computed only within each window, it does not allow for information exchange between windows. To address this limitation, Shifted Window Multi-Head Self-Attention (SW-MSA) was introduced, which shifts the windows by 4 to 9 positions. This operation facilitates the exchange of information between previously isolated windows, effectively overcoming the limitation of W-MSA, which can only capture local information.

**Fig. 6:**
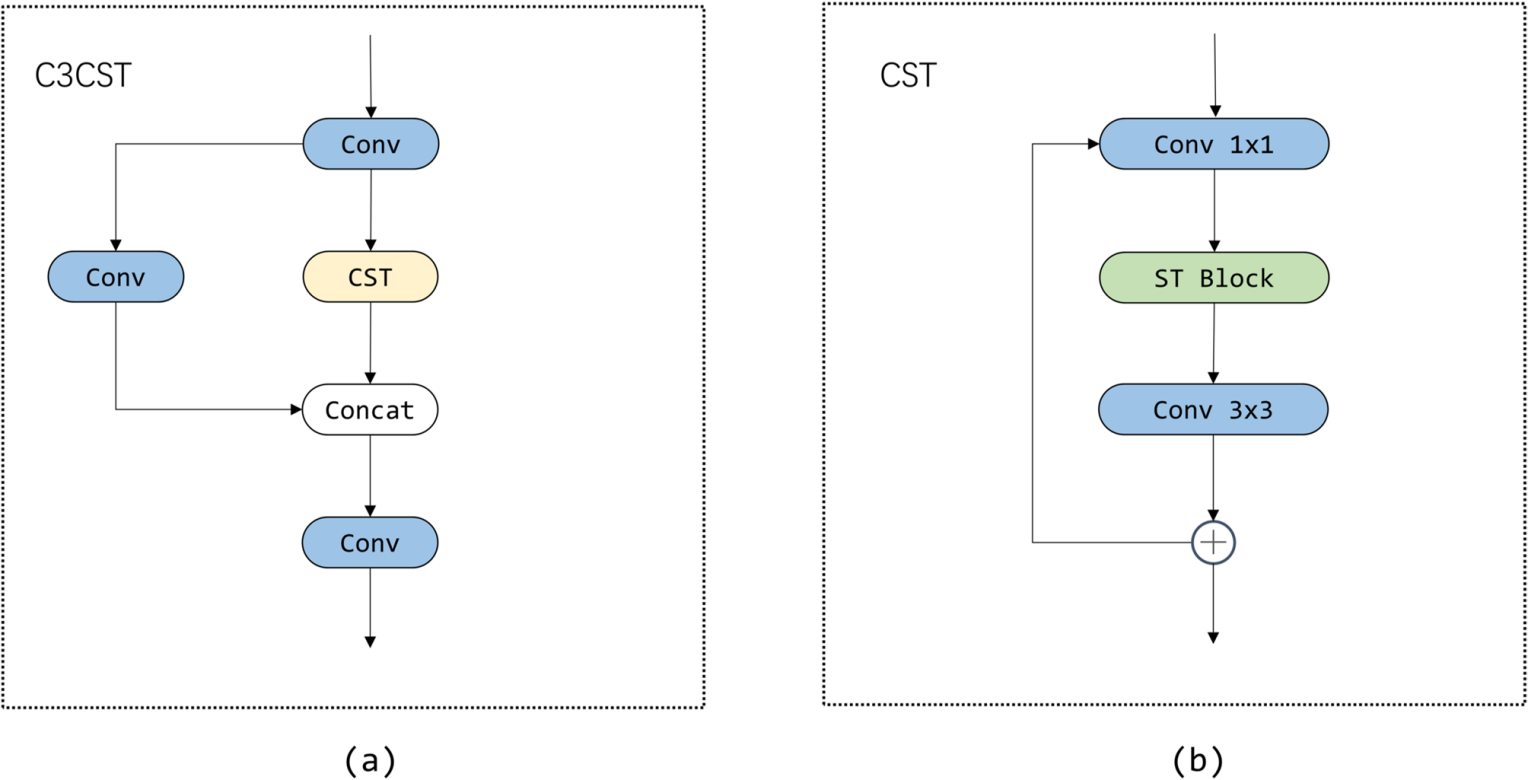
The structural composition of C3CST and CST.

Remote sensing images often feature complex backgrounds and significant variations in target size. The PANet tends to lose important feature information during the sampling process, leading to omissions and false detections, which degrade model performance. To address this issue, we designed the C3CST module. The C3CST module is primarily an improved version of the C3 module, where we integrated the CST to enhance the model’s ability to perceive both global and local information, thereby expanding the receptive field. Figure 6 illustrates the structure of the C3CST module as well as the structure of the CST module. CST module refers to the Swin Transformer module added to the Bottleneck of C3, which is added in two convolutional layers. The C3CST module replaces the Bottleneck of C3 with the CST module. This is where the module name of C3CST comes from.

The CST module is an enhancement of the Bottleneck module, achieved by inserting a Swin Transformer Block between two convolutional layers. The C3CST module is designed to improve the extraction of both local and global features, addressing the limited receptive field of the original C3 module. This module effectively mitigates the issue of excessive similarity between the target and the background in complex remote sensing images. By applying the C3CST module, the occurrence of leakage and false detections is reduced to some extent, thereby further enhancing model performance.

### Shape-IoU

Most current border loss functions focus solely on the geometric relationship between the ground truth (GT) frame and the predicted frame, calculating the loss based on the relative position and shape. However, attributes such as the shape of the frame itself also significantly impact border regression.

The impact of ground truth (GT) frame shapes on Intersection over Union (IoU) can be summarized as follows:

- Non-square GT frames with distinct long and short sides induce variations in IoU values due to differences in shape and scale between the regression sample and the GT frame, even with identical non-zero offsets and shape biases.
- For regression samples of the same scale, non-zero offsets and shape deviations cause the bounding box shape to influence IoU values, with deviations along the short side having a more pronounced effect.
- For regression samples of identical shape, smaller-scale samples exhibit greater sensitivity to GT box shape variations compared to larger-scale samples under identical non-zero offsets and shape biases.

**Fig. 7:**
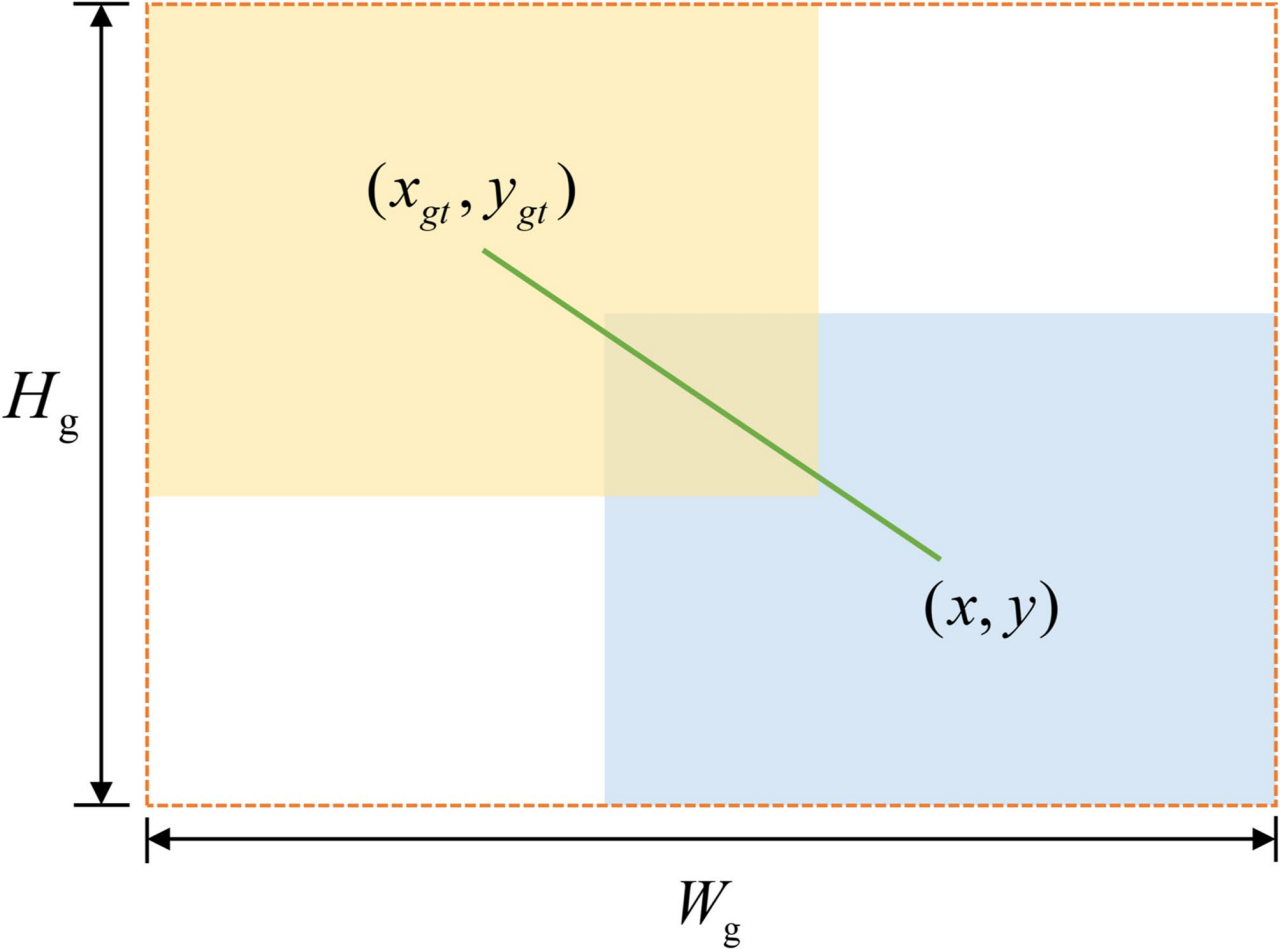
Parameter definitions for Shape-IoU loss.

To address the aforementioned issues, Shape-IoU^51^ was proposed. This method focuses on the shape and scale of the bounding box itself to compute the loss, thereby improving the accuracy of bounding box regression. Figure 7 shows the parameters of the Shape-IoU.

The most common way to express IoU (Intersection over Union) is as follows:

7 $$IoU=\frac{{\left| {B \cap {B^{gt}}} \right|}}{{\left| {B \cup {B^{gt}}} \right|}}$$

Where $B$ is the prediction box, ${B^{gt}}$ is the GT box.

To compute $distanc{e^{shape}}$ (shape-related distance loss), we first need to compute the weight coefficients for the shape and scaling, which can be calculated as follows:

8 $$ww=\frac{{2 \times {{({w^{gt}})}^{scale}}}}{{{{({w^{gt}})}^{scale}}+{{({h^{gt}})}^{scale}}}}$$

9 $$hh=\frac{{2 \times {{({h^{gt}})}^{scale}}}}{{{{({w^{gt}})}^{scale}}+{{({h^{gt}})}^{scale}}}}$$

Where ${w^{gt}}$ and ${h^{gt}}$ are the width and height of the true box, scale is the scaling factor, $ww$ and $hh$ are the weight coefficients for shape and scale.

After obtaining the weights, we calculate the value of $distanc{e^{shape}}$. Here’s his formula:

10 $$distanc{e^{shape}}=hh \times \frac{{{{({x_c} - {x_c}^{{gt}})}^2}}}{{{c^2}}}+ww \times \frac{{{{({y_c} - {y_c}^{{gt}})}^2}}}{{{c^2}}}$$

Where $c$ is the diagonal length of the minimum bounding rectangle of the predicted and true boxes. ${x_c}$ and ${y_c}$ are the center coordinate of the prediction box, $x_{c}^{{gt}}$ and $y_{c}^{{gt}}$ are the center point coordinate of the real box.

Then we need to calculate ${\Omega ^{shape}}$ (shape loss). Here is the formula for it:

11 $${\Omega ^{shape}}=\sum\nolimits_{{t=w,h}} {{{(1 - {e^{wt}})}^\theta },\theta =4}$$

Where ${\Omega ^{shape}}$ represents the shape similarity loss, $\theta$ is the is a smoothing factor used to enhance the sensitivity of the loss function to shape differences, so that the loss increases faster when the shape differences are large. ${w_t}$ stands for the width or height shape dissimilarity measure.

The calculation formula of ${w_t}$ is shown below, where ${w_w}$ stands for the width shape difference measure formula, and ${w_h}$ l stands for the height shape difference measure formula.

12 $$\left\{ \begin{gathered} {w_w}=hh \times \frac{{\left| {w - {w^{gt}}} \right|}}{{\hbox{max} (w,{w^{gt}})}} \hfill \\ {w_h}=ww \times \frac{{\left| {h - {h^{gt}}} \right|}}{{\hbox{max} (h,{h^{gt}})}} \hfill \\ \end{gathered} \right.$$

Where ${w_w}$ and ${w_h}$ measure the shape difference between the predicted and true boxes in the width and height directions, respectively.

Finally, the shape-IoU loss function is expressed as follows:

13 $${L_{Shape - IoU}}=1 - IoU+distanc{e^{shape}}+0.5*{\Omega ^{shape}}$$

## Experiments

To evaluate the performance of the model in low-resolution remote sensing image object detection, two datasets, DIOR^50^ and DOTA, are used for validation. This section outlines the experimental conditions, including the environment, setup, and evaluation metrics. Additionally, we focus on assessing the model’s performance on the two datasets, followed by an analysis of the experimental results. Finally, ablation experiments are conducted to verify the effectiveness of each module.

### Experiment environment and details

The experimental setting is orchestrated as depicted in Table 1, where all experiments were conducted under identical configurations. In the present experiment, the model is trained using the SGD optimizer with an initial learning rate of 0.01. Both the DIOR and DOTA datasets are trained for 100 epochs, with an image resolution of 640 × 640.Batch is 16. The ratio of training and validation data is split 8:2. The data augmentation parameters are as follows: mosaic:1.0; scale:0.5; hue:0.1; saturation and exposure:0.7; fliplr:0.5; degrees:10; translate:0.1, and the rest is 0. Table 1. shows the experimental environment. The baseline model in this experiment is yolov5s, and all training parameter Settings are consistent with the remote sensing image object detector Settings in this study.

**Table 1:** Experimental Environment.

Configuration	Parameters
CPU	i7-12700 F
GPU	RTX 3080ti 12GB
Operation System	Linux Ubuntu
Environment	CUDA 12.2

### Datasets

The DOTA dataset is an optical remote sensing image dataset consisting of 2,806 aerial images, each with a resolution of 4000 × 4000 pixels and spatial resolution ranging from 10 to 300 m. The dataset includes 15 object categories and 18,828 instance objects. The dataset exhibits significant variations in object scales, ranging from large-scale targets (e.g., airport runways) to small-scale targets (e.g., vehicles). This scale diversity poses a challenge to the scale invariance of detection models. The images cover diverse and complex environments, including urban areas, rural regions, coastlines, and airports. The scenes often contain cluttered backgrounds, densely distributed objects, and instances of occlusion and overlapping. Due to the large image sizes, which are too large for direct input into the network, the segmentation tool provided with YOLO is used to segment the images, resulting in images of size 1024 × 1024. The segmented images are then split into a training set and a validation set in an 8:2 ratio.

The DIOR dataset is another optical remote sensing image dataset, containing 23,463 images across 20 object categories and 192,474 instance objects. The images in the dataset have a resolution of 800 × 800 pixels and a spatial resolution ranging from 0.5 to 3 m. The dataset is split into training, validation, and test sets in a 6:2:2 ratio. Due to its variety and comprehensiveness, this dataset provides a robust platform for evaluating model performance.

### Evaluation metrics

In this experiment, performance metrics such as precision (AP), mean average precision (mAP), frames per second (FPS), and parameter count (Parameters) were employed to evaluate the detection capabilities of the model. Precision (P) and recall (R) are computed based on true positives (TP), false positives (FP), true negatives (TN), and false negatives (FN):

14 $$P=\frac{{TP}}{{TP+FP}}$$

15 $$R=\frac{{TP}}{{TP+FN}}$$

By plotting precision and recall on a precision-recall curve (P-R), the area under the curve (AP) is defined, and the mean average precision (mAP) is calculated as the mean value of AP across all categories. N is the number of all categories.

16 $$AP=\int_{0}^{1} {P(R)dR}$$

17 $$mAP=\frac{1}{N}\sum {AP}$$

### Experiment result

We evaluate the performance of our model on the DOTA v1.0 and DIOR datasets, comparing it with other representative models. The results, shown in Tables 2 and 3, demonstrate a 1.9% improvement in mAP@50 on the DIOR dataset and a 2.8% improvement in mAP@50 on the DOTA dataset compared to the baseline model, highlighting its effectiveness in remote sensing image object detection.

### Comparative performance evaluation of models on the DOTA dataset

Compared with most basic models, YOLOV5 has the best performance, so we use it as the baseline model. Table 2. presents the parameters of both the benchmark model and our model. It compares and analyzes the performance of ours with baseline on the DOTA dataset. Compared to the baseline model, the proposed model achieves consistent improvements over the baseline, with relative gains of + 1.8% in precision, + 2.1% in recall, + 2.8% in mAP@50 and + 2.3 in mAP@[0.5:0.95]. These improvements align with the initial design objectives, and all performance metrics have been improved.

**Table 2:** Performance comparison of our model and baseline on the DOTA dataset.

Method	Precision	Recall	mAP@0.5	mAP@[0.5:0.95]	Parameters	GFLPSs	FPS
baseline	75.7	64.0	67.1	40.7	7.06	16.1	85
Ours	77.5	66.1	69.9	43.0	9.52	53.4	63.8

**Fig. 8:**
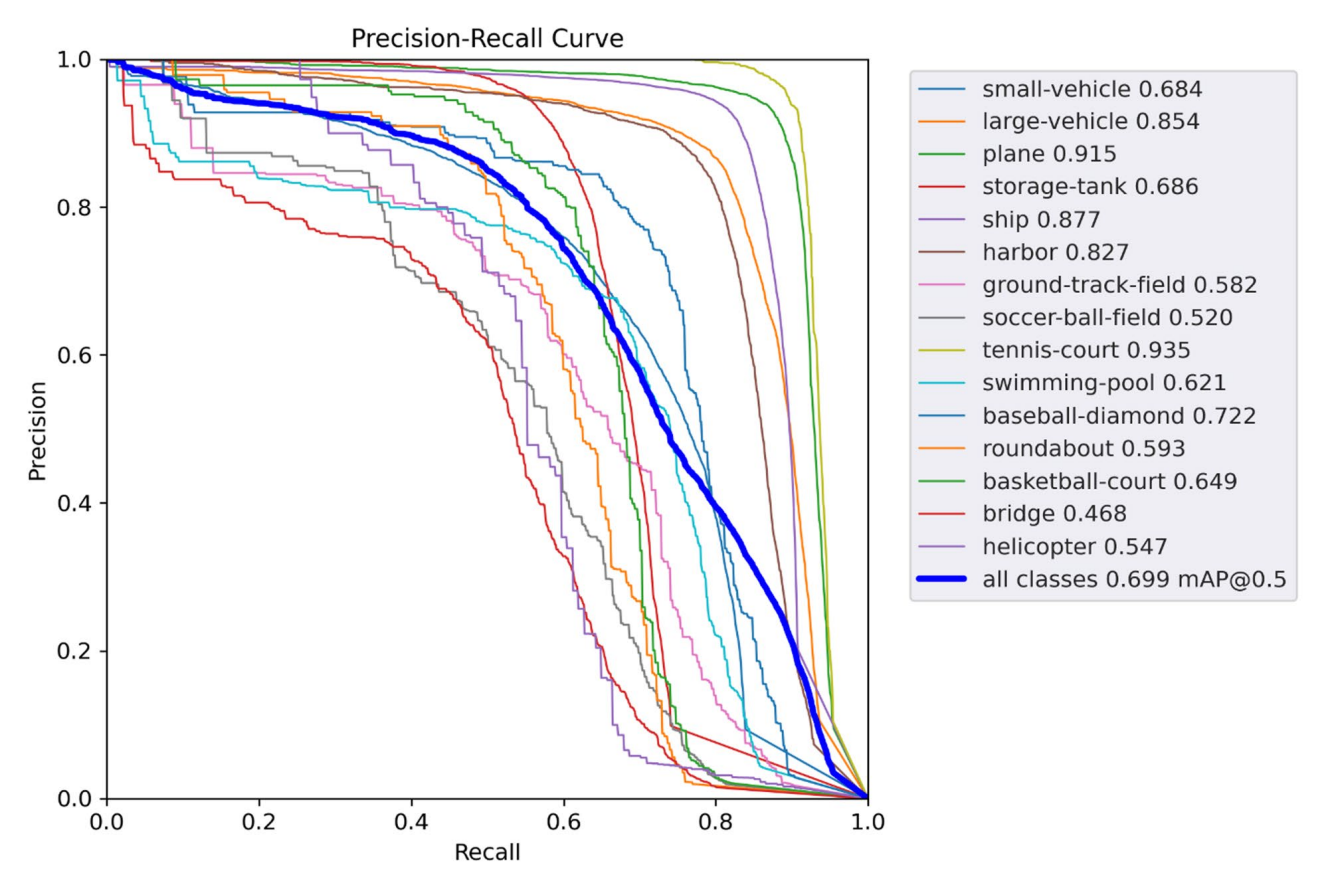
Precision-Recall curves for object categories on the DOTA test set.

Figure 8 presents the Precision-Recall (P-R) curves for all object categories in the DOTA test set, with corresponding Average Precision (AP) values annotated in the legend. The larger the AP, the better the detection performance. The results demonstrate particularly strong detection performance for structured objects, with airplanes (AP = 0.915), ships (AP = 0.877), and tennis courts (AP = 0.935) achieving the highest scores. Notably, the model also shows improved detection capability for smaller objects compared to baseline methods.

**Fig. 9:**
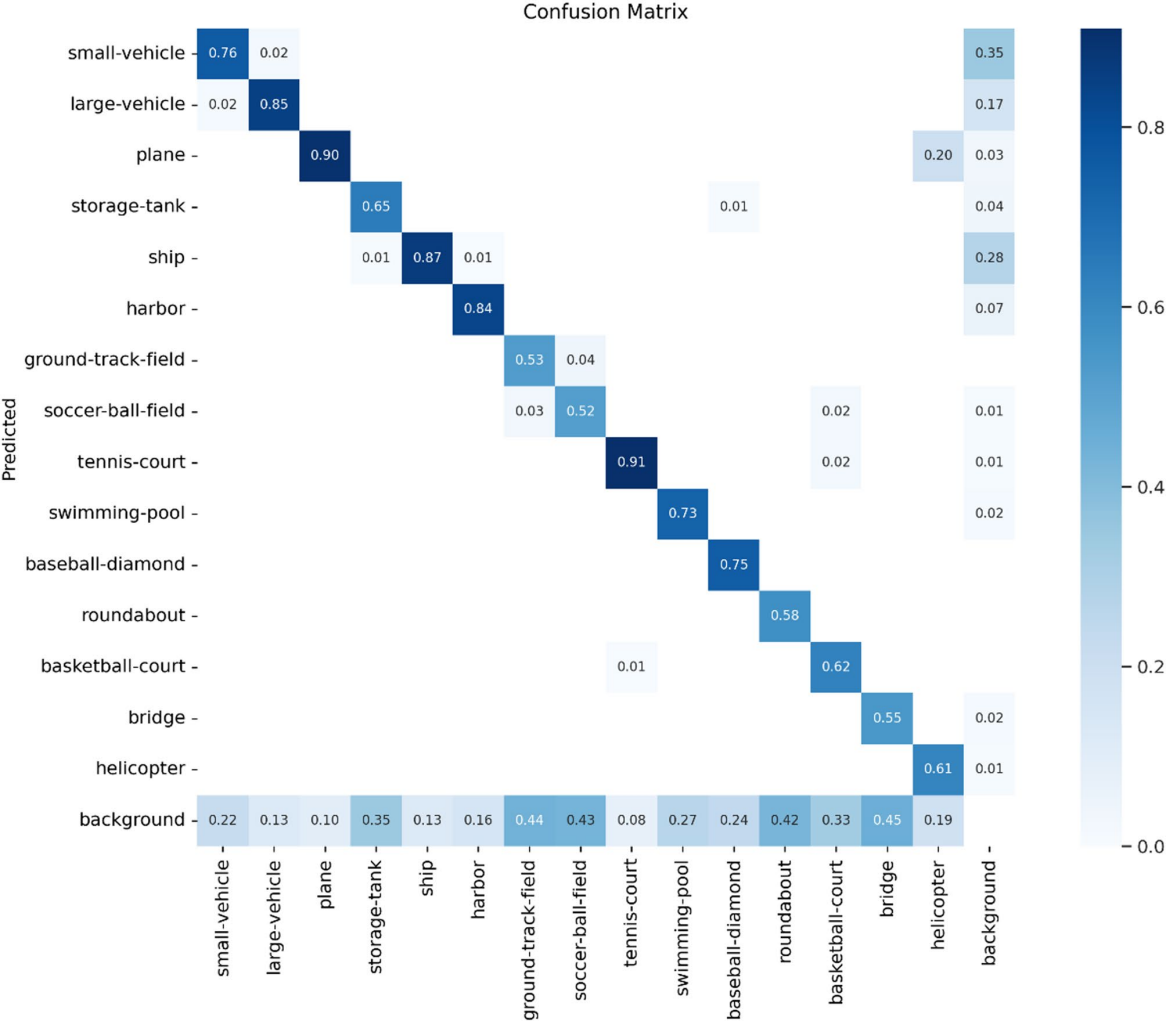
Confusion matrix for the DOTA test set.

An example of the confusion matrix is visualized in Fig. 9. It illustrates the classification performance for each category, where each row represents the predicted category, each column represents the actual category, and the diagonal values correspond to the proportion of correctly classified instances.

The confusion matrix in Fig. 9 provides further insights into category-specific performance. While diagonal values indicate strong accuracy (e.g., 93.5% for tennis courts), two error patterns are observed: Inter-class confusion: Bridges (62.1% accuracy) are frequently misclassified as overpasses (column ‘RA’), likely due to their geometric similarity in aerial imagery. Small-target homogeneity: Vehicles and ships show mutual misclassification (off-diagonal values > 15%), suggesting that further resolution enhancement is needed for sub-10px objects. These findings corroborate the P-R curve results (Fig. 8) and highlight directions for future work.

To validate the effectiveness of the model in target detection, a comparative test was conducted on the DOTA dataset. The results are presented in Table 3. When compared to LO-DET^53^A2S-DET^54^R2CNN^55^D-YOLO^56^ICN^57^YOLO-Remote^70^ and FFCA-YOLO^71^our model demonstrates significant improvements. The 2.8% improvement over the baseline model, along with improvements in object classification across all categories, highlights the model’s effectiveness. The best detection method for each object is indicated in black font.

**Table 3:** Performance comparison of different models on the DOTA dataset.

Method	mAP	PL	BD	BR	GTF	SV	LV	SH	TC	BC	ST	SBF	RA	HA	SP	HC
RADET	69.1	79.5	**77.0**	48.1	65.8	65.5	74.4	68.9	89.7	78.1	75.0	49.9	64.6	66.1	**71.6**	**62.2**
LO-DET	66.7	89.2	66.1	31.3	56.0	70.1	71.0	84.3	90.7	75.1	**81.3**	44.7	59.3	60.0	65.1	48.4
R2CNN	60.7	81.0	65.7	35.3	67.4	60.0	50.9	55.8	90.7	66.9	72.4	**55.1**	52.2	55.1	53.4	48.2
D-YOLO	68.1	86.6	71.4	**54.6**	52.5	**79.2**	70.6	**87.8**	82.2	54.1	75.0	51.0	**69.2**	66.4	59.2	51.3
ICN	68.2	81.4	74.3	47.7	**70.3**	64.9	67.8	70.0	90.8	**79.1**	78.2	53.6	62.9	67.0	64.2	50.2
YOLO-Remote	69.0	92.1	73.9	53.1	59.2	70.3	82.1	87.7	83.6	60.1	69.1	50.3	58.0	81.1	62.2	53.1
FFCA-YOLO	63.0	89.4	64.9	36.2	35.5	67.1	84.5	88.0	91.3	54.4	71.5	40.7	48.3	78.1	60.9	33.7
YOLOV5S	67.1	91.0	70.4	42.8	55.6	67.6	84.3	87.0	92.5	59.6	66.6	48.2	55.4	81.6	60.7	42.9
Ours	**69.9**	**91.5**	72.2	46.8	58.2	68.4	**85.4**	87.7	**93.5**	64.9	68.6	52.0	59.3	**82.7**	62.1	54.7

**Fig. 10:**
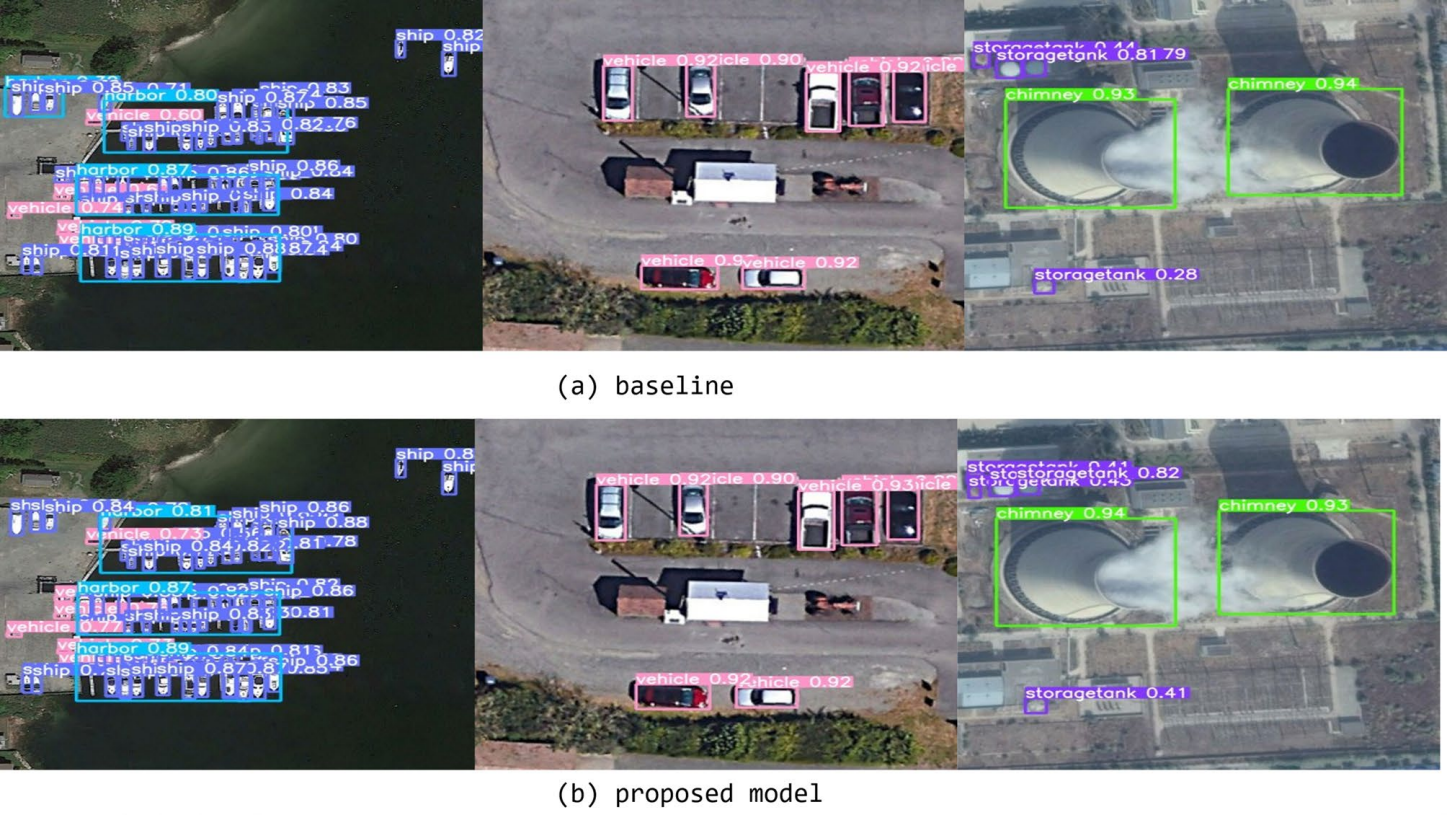
Qualitative comparison between baseline (**a**) and proposed model (**b**) on a DOTA sample. Boxes with different colors represent the recognition of different objects.

To visualize the performance difference, we compare target detection on the same image. Figure 10. presents the comparison between the two methods. The object boxes with different colors represent different object categories, and the top left corner has the name and the confidence score written. In the two comparison figures, it is evident that ships and ports can be accurately identified even in high-density harbor areas, demonstrating a notable improvement in detection precision. The model also shows enhanced performance in detecting vehicles in low-density scenarios. Moreover, in images containing objects of varying sizes, our model successfully detects targets in previously missed areas without compromising the recognition of surrounding objects. This indicates the model’s strong capability to handle both dense and complex scenes with mixed object scales. By looking at the object recognition and confidence scores of the three sets of images, the improvement in model performance can be clearly seen.

Low-resolution remote sensing image means that the resolution of the image entering the object detector is lower than its original size. It is well known that the higher the image resolution, the better the performance of the model after training. However, it needs to improve the hardware and increase the training speed. In this experiment, it is stipulated that all images below 640 × 640 are low-resolution remote sensing images. We will compare the performance of the trained model between the original resolution and the low resolution.

As shown in Table 4, we first list the experimental data and results of the original size in the baseline model, and then list the performance of the model in the baseline model with low resolution images, and the last experiment with low resolution in the remote sensing image object detection we designed. As you can see from the Table 4, we get better performance by using less memory on the graphics card.

**Table 4:** Performance at different resolutions on the DOTA dataset.

Method	Resolution	Precision	Recall	mAP@0.5	GPU_men
Baseline	1024 × 1024	75.8	66.5	69.1	7.34G
Baseline	640 × 640	75.7	64.0	67.1	5.21G
Ours	640 × 640	77.5	66.1	69.9	6.03G

### Comparative performance evaluation of models on the DIOR dataset

The model was initially designed to address the challenges posed by small targets and complex background targets. Since the distribution of target sizes in the DIOR dataset is uniform, we performed target detection using our model to evaluate the model’s generalization ability. The comparison results are presented in Table 5. We compared our model with CANet^58^TRD^59^FSoD-Net^60^MFPNet^61^MDCT^62^CF2SPN^63^MAS-YOLO^64^YOLO-Remote and FFCA-YOLO. The experimental results demonstrate that SSE-YOLO exhibits strong generalization capabilities, improving performance by 1.9%.

**Table 5:** Performance comparison of different models on the DIOR dataset. Ablation Experiments.

Method	mAP	Parameters
CANet	74.3	47.02
TRD	66.8	13.7
FSoD-Net	71.8	232.3
MFPNet	71.2	45.11
CF2PN	67.3	91.6
MDCT	80.5	9.6
MAS-YOLO	79.6	6.91
YOLO-Remote	73.4	3.12
FFCA-YOLO	70.5	2.34
YOLOv5s	83.7	7.06
Ours	85.5	9.52

To validate the effectiveness of individual our model, we conducted ablation experiments on the DOTA dataset and analyzed the results. We began by testing the individual network modules replaced in the baseline to evaluate their performance. Ablation experiments were then performed to further analyze the contributions of each module.

As shown in Table 6, in the first experiment, we evaluated the detection performance of the baseline model on the DOTA dataset. The second experiment replaced the original CIoU loss function with the Shape-IoU loss function, resulting in a modest improvement in recall and average precision. In the third experiment, The downsampling network was replaced with FEDNet. The fourth experiment replaced thePANet with STPANet. Subsequently, we progressively integrated the various network modules and analyzed their combined performance results.

**Table 6:** The baseline combining different modules on the DOTA dataset.

Shape-IoU	FEDNet	STPANet	Precision	Recall	mAP@0.5
			75.7	64.0	67.1
√			75.5	65.0	67.8(+0.7)
	√		76.6	65.9	69.2(+2.1)
		√	75.9	64.6	68.1(+1.0)
√	√		76.9	66.1	69.5(+2.4)
	√	√	77.0	65.4	69.4 (+ 2.3 )
√		√	76.1	65.7	68.3 (+ 1.2 )
√	√	√	77.5	66.1	69.9(+2.8)

Ablation experiments were conducted on the SPDConv and ECA modules within FEDNet to evaluate their individual and combined contributions. The first experiment utilized the baseline model without any modifications. In the second experiment, the standard downsampling convolution was replaced with SPDConv. The third experiment introduced only the ECA module, and the fourth combined both SPDConv and ECA to assess the effect of their joint integration. Table 7 presents the ablation results on the DOTA dataset.

**Table 7:** The effects of the SPDCov and ECA on the DOTA dataset.

Method	Precision	Recall	mAP@0.5
None	75.7	64.0	67.1
SPDConv	76.6	65.7	68.6
ECA	75.7	64.5	67.7
SPDConv + ECA	76.6	65.9	69.2

## Conclusions

This paper proposes an object detection model that integrates Fine-Grained Information Enhancement with a Swin Transformer architecture. The incorporation of the Shape-IoU loss enables the model to focus more effectively on the intrinsic attributes of bounding boxes during regression, thereby accelerating convergence. Unlike traditional loss functions that prioritize positional offset, Shape-IoU emphasizes shape alignment, which—when paired with a suitable scale parameter—significantly enhances small object detection, yielding a 0.7% increase in mAP compared to the baseline.

Within FEDNet, SPDConv combines dilated convolutions with downsampling to preserve fine-grained information and mitigate the feature loss typically associated with conventional methods, resulting in a 1.8% mAP improvement. However, the increased channel dimensions introduced by SPDConv can lead to substantial information loss during compression. To address this, the ECA module is integrated to adaptively reweight channel features without introducing additional computational overhead, thereby enhancing the network’s representational capacity.

Moreover, the limited performance of the original C3 module on small targets—especially evident in the DOTA dataset—was improved by embedding Swin Transformer blocks, which expand the receptive field and reduce the likelihood of missed or incorrect detections. With all modules incorporated, the proposed model achieves a 2.8% performance gain over the baseline on the DOTA dataset. Additionally, the model demonstrates strong generalization capability, achieving a 1.5% mAP improvement on the DIOR dataset. The ablation studies further validate the individual and collective contributions of each component, confirming their effectiveness in enhancing overall detection performance.

**Fig. 11:**
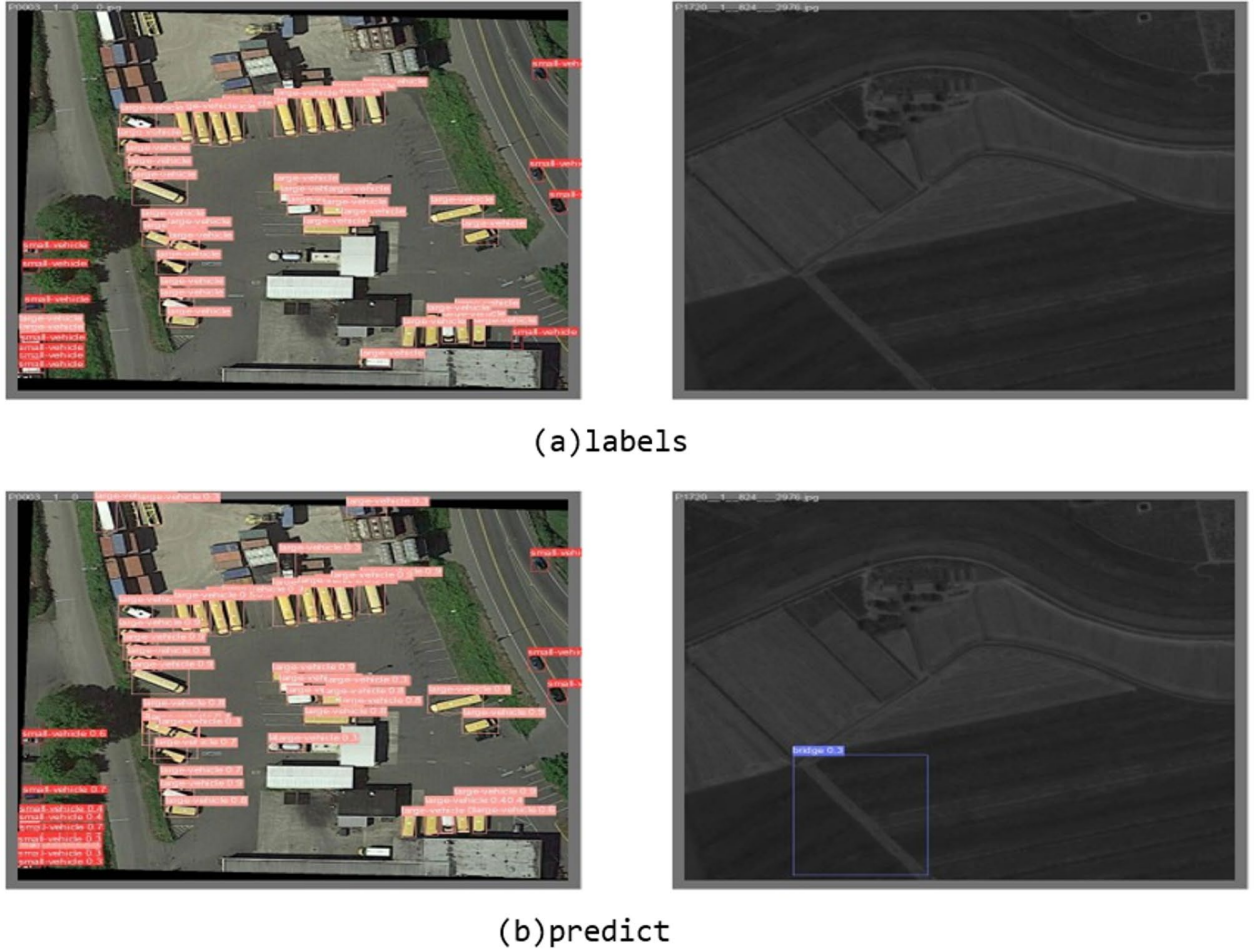
Fault case analysis.

Although the proposed method demonstrates notable improvements in remote sensing image object detection, several limitations remain. For example, relying on horizontal bounding boxes adversely affects the detection of overlapping objects, resulting in missed or incorrect detections. As illustrated in Fig. 11, noticeable deviations can be observed between the predicted bounding boxes and the ground truth labels. This discrepancy may be attributed to visual similarity in object colors and the loss of discriminative features caused by downsampling in FEDNet. Future research will focus on addressing such limitations by improving model design to better distinguish overlapping and visually similar objects.

The performance gains of the proposed remote sensing object detector are primarily achieved at the expense of increased training time, higher GFLOPs, and a larger number of model parameters. This increase in computational complexity is largely due to the incorporation of SPDConv and Swin Transformer modules within FEDNet. Future work will focus on enhancing model performance while maintaining a balance between lightweight design and detection accuracy, enabling deployment in real-time remote sensing detection systems.

## Data Availability

The data used in this paper is publicly available and can be accessed at https://captain-whu.github.io/DOTA/index.html and https://aistudio.baidu.com/datasetdetail/258221.
